# Quinine Pill Mass—Formulæ

**Published:** 1874-08

**Authors:** H. M. Smith


Quinine Pill Mass—*By Dr. H. M. Smith.*—The following is the formula:

| | | |
|---|---|---|
| ℞.—Sulph. Quinine, | . . . . . . | 30 gr. |
| Tartaric Acid, | . . . . . . . . | 3 gr. |
| Simple Syrup, | . . . . . . . | 5 drops. |

Mix acid and syrup, then add quinia gradually, and work with spatula on a slab until mass is formed. This mass makes a beautiful, compact and small pill, when each one contains two grains of quinine.

In connection with this, permit me to give you another formula containing quinine:

| | | |
|---|---|---|
| ℞.—Sulph. Quinine, | . . . . . . | 15 grs. |
| Tannic Acid, | . . . . . . . | 5 grs. |
| Comp. Spts. Lavender, | . . . . . | 2 ʒ. |
| Simple Syrup, | . . . . . . . | 2 ℥. |

Mix the quinine and tannin in a mortar, add a small portion of the syrup and then the lavender, and, after stirring well, add balance of the syrup. It can be easily made by grinding up white sugar, quinine and tannin, and then adding lavender and water enough to make two ounces of syrup. I prefer this mode of mixing, when time permits, to using the syrup already prepared. I am aware that it has been said that the quinine and tannin form an insoluble compound, but a twenty years' experience convinces me of the falsity of the assertion. I have thought the preparation more potent than the simple sulphate. Any child will take this mixture readily, and many will ask for and take it as they would so much molasses.—*The Clinic—July.*

